# RETRACTION: Island Sternocleidomastoid Myocutaneous Flap for Posterior Pharyngeal Wall Defect Repair After Anterior Cervical Spine Surgery

**DOI:** 10.1111/iwj.70654

**Published:** 2025-04-23

## Abstract

**RETRACTION:** ZhangX. , ZhaoR. , WangG. , ChenY. , DingP. , YangX. , ZhaoZ. , and ZhangY. , “Island Sternocleidomastoid Myocutaneous Flap for Posterior Pharyngeal Wall Defect Repair After Anterior Cervical Spine Surgery,” International Wound Journal 19, no. 1 (2022): 169–177, 10.1111/iwj.13612.33999495 PMC8684854

The above article, published online on 17 May 2021, in Wiley Online Library (http://onlinelibrary.wiley.com/), has been retracted by agreement between the journal Editor in Chief, Professor Keith Harding; and John Wiley & Sons Ltd. Following an investigation by the publisher, all parties have concluded that this article was accepted solely on the basis of a compromised peer review process. The editors have therefore decided to retract the article. The authors did not respond to our notice regarding the retraction.

**RETRACTION:** X. Zhang , R. Zhao , G. Wang , Y. Chen , P. Ding , X. Yang , Z. Zhao , and Y. Zhang , “Island Sternocleidomastoid Myocutaneous Flap for Posterior Pharyngeal Wall Defect Repair After Anterior Cervical Spine Surgery,” International Wound Journal 19, no. 1 (2022): 169–177, 10.1111/iwj.13612.33999495 PMC8684854

The above article, published online on 17 May 2021, in Wiley Online Library (http://onlinelibrary.wiley.com/), has been retracted by agreement between the journal Editor in Chief, Professor Keith Harding; and John Wiley & Sons Ltd. Following an investigation by the publisher, all parties have concluded that this article was accepted solely on the basis of a compromised peer review process. The editors have therefore decided to retract the article. The authors did not respond to our notice regarding the retraction.

